# Impact of social determinants of health on ATN biomarkers and neurodegeneration in the ReDLat cohort

**DOI:** 10.1002/alz70856_106516

**Published:** 2026-01-10

**Authors:** Matias Pizarro, Ariel Caviedes, Joaquín Migeot, Hernan Hernandez, Felipe Cabral‐Miranda, Nickole P. Marin‐Díaz, Carolina Gonzalez‐Silva, Andrea Slachevsky, Hernando Santamaria‐Garcia, María Isabel Behrens, Diana L Matallana, David Aguillon, Nilton Custodio, Martin Alejandro Bruno, José Alberto Avila Funes, Agustin Ibanez, Claudia Duran‐Aniotz

**Affiliations:** ^1^ Center for Social and Cognitive Neuroscience (CSCN), School of Psychology, Universidad Adolfo Ibanez, Santiago, Chile., Santiago, Región Metropolitana, Chile; ^2^ Latin American Brain Health Institute (BrainLat), Universidad Adolfo Ibanez, Santiago, Región Metropolitana, Chile; ^3^ Universidad Adolfo Ibañez, Santiago, Chile; ^4^ Latin American Brain Health Institute (BrainLat), Universidad Adolfo Ibañez, Santiago, Chile; ^5^ Global Brain Health Institute (GBHI), University of California San Francisco (UCSF); & Trinity College Dublin, Dublin, Leinster, Ireland; ^6^ Federal Univesity of Rio de Janeiro, Rio de Janeiro, Rio de Janeiro, Brazil; ^7^ Center for Social and Cognitive Neuroscience (CSCN), School of Psychology, Universidad Adolfo Ibanez., Santiago, Región Metropolitana, Chile; ^8^ Cognitive Neurology and Dementia, Neurology Department, Hospital del Salvador, Santiago, Chile; ^9^ Neurology Department, Hospital del Salvador, University of Chile, Santiago, Región Metropolitana de Santiago, Chile; ^10^ Pontificia Universidad Javeriana, Bogotá, Colombia; ^11^ Global Brain Health Institute (GBHI), University of California San Francisco (UCSF); & Trinity College Dublin, San Francisco, CA, USA; ^12^ Centro de Investigación Clínica Avanza (CICA), Hospital Clínico Universidad de Chile, Santiago, Chile; ^13^ Hospital Clínico de la Universidad de Chile, Santiago de Chile, Chile; ^14^ Hospital Universitario San Ignacio, Bogotá, Bogotá, Colombia; ^15^ Neurosciences Group of Antioquia, University of Antioquia, Medellín, Colombia; ^16^ Unit Cognitive Impairment and Dementia Prevention, Peruvian Institute of Neurosciences, Lima, Peru, Lima, Lima, Peru; ^17^ Instituto de Ciencias Biomédicas (ICBM) Facultad de Ciencias Médicas, Universidad Catoóica de Cuyo, San Juan, San Juan, Argentina; ^18^ Instituto Nacional de Ciencias Médicas y Nutrición Salvador Zubirán, México, DF, Mexico; ^19^ Global Brain Health Institute (GBHI), University of California San Francisco (UCSF); & Trinity College Dublin, Dublin, Ireland

## Abstract

**Background:**

Social determinants of health (SDH), including subjective socioeconomic status (SES), education, nutrition, and traumatic life events, shape neurodegenerative risk by influencing plasma AT(N) biomarkers (Aβ40, Aβ42, Aβ42/Aβ40 ratio, *p*‐tau181, and *p*‐tau217). These indicators reflect pathological processes in Alzheimer's disease (AD) and frontotemporal lobar dementia (FTLD). While the link between SDH and dementia is increasingly recognized, little is known about how SDH affects AT(N) biomarkers in diverse populations, particularly in regions with marked inequalities like Latin America. This study examined the ReDLat cohort to explore associations between SDH and AT(N) biomarkers in cognitively normal (CN) individuals and those with AD or FTLD.

**Method:**

ReDLat participants from several Latin American countries were assessed for subjective SES, education, nutrition, basic needs, and traumatic life events. Plasma levels of Aβ40, Aβ42, the Aβ42/Aβ40 ratio, *p*‐tau181, and *p*‐tau217 were measured. Multiple regression models (adjusted for age and sex) investigated associations between SDH factors and biomarker levels. Analyses were stratified by diagnostic group (CN, AD, FTDL) to identify which SDH factors correlated most strongly with biomarker alterations and to determine whether these relationships varied by cognitive status.

**Result:**

Subjective SES and traumatic events showed positive associations with increased Aβ40, Aβ42, and *p*‐tau181 levels in all participants. Lower SES was linked to a reduced Aβ42/Aβ40 ratio, indicating potential heightened cerebral Aβ42 deposition, notably in AD. Education exhibited a protective role: higher educational attainment positively correlated with *p*‐tau217 in CN participants. Conversely, financial adversity was linked to elevated *p*‐tau217 in FTDL patients, suggesting distinct SDH influences by dementia subtype. Nutrition emerged as a key determinant, strongly associating with Aβ40 in AD and with the Aβ42/Aβ40 ratio in CN individuals.

**Conclusion:**

These findings highlight the significant impact of SDH on AT(N) biomarker profiles, underscoring social inequality's contribution to neurodegenerative risk. Lower SES, poorer nutrition, and limited education were strongly tied to unfavorable biomarker changes, emphasizing the need for targeted interventions addressing SDH disparities. Future longitudinal studies should clarify the mechanisms underlying these associations to better inform dementia prevention strategies. By addressing these modifiable SDH factors, public health initiatives may help reduce dementia risk across diverse populations worldwide. Additional research is warranted.

